# Regulation of Tocopherol Biosynthesis During Fruit Maturation of Different *Citrus* Species

**DOI:** 10.3389/fpls.2021.743993

**Published:** 2021-10-06

**Authors:** Florencia Rey, Lorenzo Zacarias, María Jesús Rodrigo

**Affiliations:** Departamento de Biotecnología de Alimentos, Instituto de Agroquímica y Tecnología de Alimentos, Consejo Superior de Investigaciones Científicas, Valencia, Spain

**Keywords:** tocopherol, vitamin E, *Citrus*, fruit, ripening, tocopherol gene expression

## Abstract

Tocopherols are plant-derived isoprenoids with vitamin E activity, which are involved in diverse physiological processes in plants. Although their biosynthesis has been extensively investigated in model plants, their synthesis in important fruit crops as *Citrus* has scarcely been studied. Therefore, the aim of this work was to initiate a physiological and molecular characterization of tocopherol synthesis and accumulation in *Citrus* fruits during maturation. For that purpose, we selected fruit of the four main commercial species: grapefruit (*Citrus paradisi*), lemon (*Citrus limon*), sweet orange (*Citrus sinensis*), and mandarin (*Citrus clementina*), and analyzed tocopherol content and the expression profile of 14 genes involved in tocopherol synthesis during fruit maturation in both the flavedo and pulp. The selected genes covered the pathways supplying the tocopherol precursors homogentisate (HGA) (*TAT1* and *HPPD*) and phytyl pyrophosphate (PPP) (*VTE5*, *VTE6*, *DXS1* and *2*, *GGPPS1* and *6*, and *GGDR*) and the tocopherol-core pathway (*VTE2*, *VTE3a*, *VTE3b*, *VTE1*, and *VTE4*). Tocopherols accumulated mainly as α- and γ-tocopherol, and α-tocopherol was the predominant form in both tissues. Moreover, differences were detected between tissues, among maturation stages and genotypes. Contents were higher in the flavedo than in the pulp during maturation, and while they increased in the flavedo they decreased or were maintained in the pulp. Among genotypes, mature fruit of lemon accumulated the highest tocopherol content in both the flavedo and the pulp, whereas mandarin fruit accumulated the lowest concentrations, and grapefruit and orange had intermediate levels. Higher concentrations in the flavedo were associated with a higher expression of all the genes evaluated, and different genes are suitable candidates to explain the temporal changes in each tissue: (1) in the flavedo, the increase in tocopherols was concomitant with the up-regulation of *TAT1* and *VTE4*, involved in the supply of HGA and the shift of γ- into α-tocopherol, respectively; and (2) in the pulp, changes paralleled the expression of *VTE6*, *DXS2*, and *GGDR*, which regulate PPP availability. Also, certain genes (i.e., *VTE6*, *DXS2*, and *GGDR*) were co-regulated and shared a similar pattern during maturation in both tissues, suggesting they are developmentally modulated.

## Introduction

Tocopherols are lipid-soluble isoprenoids of the tocochromanol family which are mainly synthesized in photosynthetic organisms (Fritsche et al., 2017; Mène-Saffrané, 2017). Their chemical structure consists of a polar chromanol ring, originated from homogentisate (HGA), and a lipophilic isoprenoid side chain derived from a specific prenyl pyrophosphate donor. While HGA is the common precursor for all tocochromanols, the polyprenyl precursor varies according to the type of tocochromanol and is phytyl pyrophosphate (PPP) for tocopherol synthesis (Mène-Saffrané, 2017). Additionally, according to the position and degree of methylation of the chromanol ring, four natural sub-forms can exist: δ- (one methyl group), β- and γ- (two methyl groups), and α-tocopherol (three methyl groups). Tocopherols are of great importance because, together with tocotrienols, they are the only natural compounds exhibiting vitamin E activity in animal cells and are essential as dietary nutrients (Traber and Sies, 1996). In plants, tocopherols play diverse physiological functions, of which their role as antioxidants, either scavenging peroxyl radicals or quenching reactive oxygen species, is probable the most notable (Havaux et al., 2005; Mène-Saffrané et al., 2010). Nonetheless, other functions have been recently described for tocopherols in plants, including their involvement in photo-assimilate transport, carbohydrate metabolism, cellular signaling and plant’s response to biotic and abiotic stresses (Falk and Munné-Bosch, 2010; Muñoz and Munné-Bosch, 2019; Ma et al., 2020b).

**FIGURE 1:**
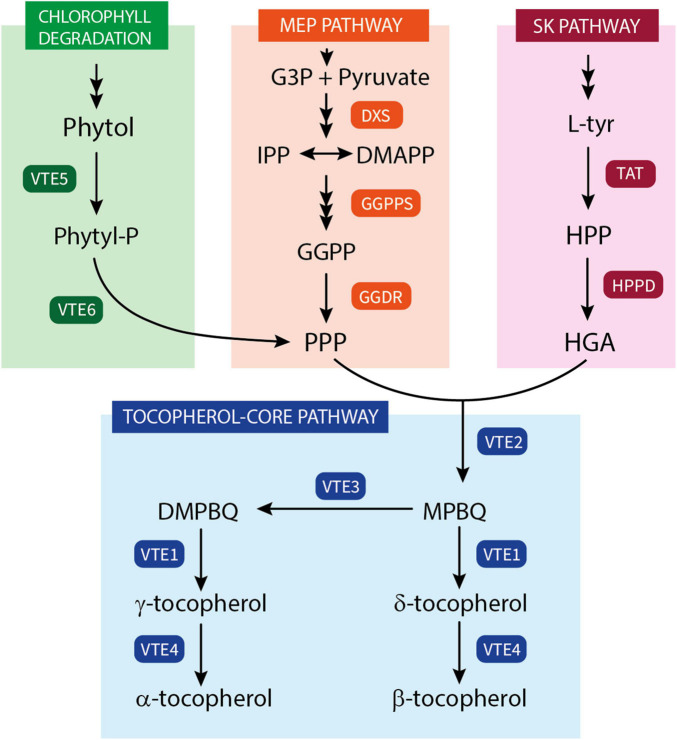
Schematic representation of tocopherol biosynthetic pathway in plants. Phytyl-P, phytyl phosphate; G3P, glyceraldehyde 3-phosphate; IPP, isopentenyl diphosphate; DMAPP, dimethylallyl diphosphate; GGPP, geranylgeranyl diphosphate; PPP, phytyl pyrophosphate; L-Tyr, amino acid L-tyrosine; HPP, 4-hydroxyphenylpyruvate; HGA, homogentisate; MPBQ, 2-methyl-6-phytyl-1,4-benzoquinol; DMPBQ, 2,3-dimethyl-6-phytyl-1,4-benzoquinol; VTE5, phytol kinase; VTE6, phytyl-P kinase; DXS, 1-deoxy-D-xylulose-5-phosphate synthase; GGPPS, GGPP synthase; GGDR, GGPP reductase; TAT, tyrosine aminotransferase; HPPD, HPP dioxygenase; VTE2, homogentisate phytyltransferase (HPT); VTE3, MPBQ methyltransferase (MPBQ-MT); VTE1, tocopherol cyclase (TC); VTE4, tocopherol methyltransferase (γ-TMT).

The tocopherol biosynthesis pathway (Figure 1) has been well-characterized in the last decades, with all the vitamin E biosynthetic genes (VTE genes) encoding the enzymes catalyzing the core steps of tocopherol synthesis identified (Fritsche et al., 2017; Mène-Saffrané, 2017; Muñoz and Munné-Bosch, 2019). Tocopherol synthesis is initiated by the condensation of HGA with PPP, a reaction regulated by homogentisate phytyl transferase (HPT; *VTE2*) that results in the formation of 2-methyl-6-phytyl-1,4-benzoquinol (MPBQ) (Collakova and DellaPenna, 2001). After this step, the pathway can split into two branches depending on the subsequent reaction of MPBQ, leading toward the synthesis of δ- and β-tocopherol or γ- and α-tocopherol. Then, MPBQ can either be directly cyclized into δ-tocopherol, by the tocopherol cyclase (TC; *VTE1*), or it can be first methylated into 2,3-dimethyl-6-phytyl-1,4-benzoquinol (DMPBQ), by a MPBQ methyltransferase (MPBQ-MT; *VTE3*). The resulting product DMPBQ is then converted into γ-tocopherol by the same TC (*VTE1*) mentioned before. The final step of tocopherol synthesis is the methylation of δ- and γ-tocopherol into β- and α-tocopherol, respectively, which is catalyzed by the γ-tocopherol methyltransferase (γ-TMT; *VTE4*). These steps represent the tocopherol-core pathway, and of these enzymes only HPT (*VTE2*) is considered exclusive to tocopherol synthesis, while MPBQ-MT (*VTE3*), TC (*VTE1*), and γ-TMT (*VTE4*) are also involved in the synthesis of the other tocochromanols (Fritsche et al., 2017; Mène-Saffrané, 2017). *VTE2* has been reported to be a limiting step in tocopherol synthesis in seeds and leaves of Arabidopsis and other model plants (Savidge et al., 2002; Collakova and DellaPenna, 2003a). However, it does not seem to limit tocopherol synthesis in fruit of species such as tomato, olive, and mandarin (Quadrana et al., 2013; Georgiadou et al., 2019; Rey et al., 2021a), where *VTE3* appears to play a more important role regulating tocopherol content (Quadrana et al., 2013; Rey et al., 2021a).

Tocopherol content in plant cells is also highly dependent on the availability of the precursors HGA and PPP (Mène-Saffrané, 2017; Pellaud and Mène-Saffrané, 2017). In plants, the precursor HGA originates in a two-step reaction from the degradation of the amino acid L-tyrosine (L-Tyr) synthetized in the shikimate (SK) pathway. L-Tyr is converted into 4-hydroxyphenylpyruvate (HPP) by a tyrosine aminotransferase (TATs), and then transformed into HGA by a HPP dioxygenase (HPPD) (Figure 1). The loss of function of either *TAT* or *HPPD* in Arabidopsis has resulted in a reduction of tocopherol levels (Norris et al., 1998; Riewe et al., 2012), while the overexpression of these genes only modestly increased total tocopherols in leaves and seeds (Tsegaye et al., 2002; Karunanandaa et al., 2005). In fleshy fruit, such as tomato and mango, the role of *HPPD* in tocopherol accumulation has been reinforced, as higher accumulation of *HPPD* transcripts has been associated with genotypes containing high tocopherols levels (Quadrana et al., 2013; Singh et al., 2017). Recently, it has been shown that engineering the chorismate–tyrosine pathway in tomato fruit to produce HPP in combination with the overexpression of Arabidopsis *HPPD* resulted in a moderate increment in tocopherols (Burgos et al., 2021). The other precursor necessary for tocopherol synthesis, PPP, can be derived from geranylgeranyl pyrophosphate (GGPP), produced in the MEP pathway, or alternatively from the recycling of phytol formed in the degradation of chlorophylls (Figure 1). Two enzymes are involved in the supply of PPP *via* the recycling of phytol: phytol kinase (*VTE5*) and phytyl phosphate kinase (*VTE6*) (Valentin et al., 2006; vom Dorp et al., 2015). VTE5 appears to be a key enzyme regulating tocopherol content in fruits of tomato and olive, controlling the supply of PPP toward the tocopherol-core pathway (Georgiadou et al., 2015; Almeida et al., 2016). Nonetheless, in ripe tomato and mandarin fruits, tocopherol accumulation appears to be more influenced by the up-regulation of genes of the MEP pathway, such as *DXS* and *GGDR* (Quadrana et al., 2013; Almeida et al., 2015; Gramegna et al., 2019; Rey et al., 2021a). While the gene *DXS* regulates the influx into the MEP pathway, *GGDR* controls the final reduction of GGPP into PPP, and therefore its final availability for condensation with HGA (Estévez et al., 2001; Pellaud and Mène-Saffrané, 2017).

Tocopherol accumulation has been mainly studied in leaves and seeds, but they have also been detected in fruits, stems, roots, flowers, and other plant tissues (Horvath et al., 2006a). In general, α-tocopherol is the main form found in leaves, while γ-tocopherol is the predominant in seeds of most species (Horvath et al., 2006a). In fruits, α-tocopherol seems to be the main isoform accumulated (Chun et al., 2006; Horvath et al., 2006a), with variable contents depending on the species and through fruit ripening (Osuna-García et al., 1998; Almeida et al., 2011; Quadrana et al., 2013; Georgiadou et al., 2015, 2019). In recent years, great advances have been made into the regulation of tocopherol biosynthesis in fruit of different species, such as tomato (Almeida et al., 2011, 2016; Quadrana et al., 2013; Gramegna et al., 2019; Burgos et al., 2021), pepper (Arango and Heise, 1998; Koch et al., 2002), olive (Georgiadou et al., 2015, 2016, 2019), and mango (Singh et al., 2017). These studies concluded that tocopherol accumulation in fruit is mainly transcriptionally regulated, and that tocopherol biosynthetic genes are modulated in a temporal manner, and also influenced by environmental factors (Almeida et al., 2011, 2020; Quadrana et al., 2013; Georgiadou et al., 2016, 2019; Singh et al., 2017; Gramegna et al., 2019).

*Citrus* is one of the world’s most important fruit crops, being highly demanded for fresh consumption and juice processing (Spreen et al., 2020). This genus is characterized for its genotypic and phenotypic diversity (Wu et al., 2018), with a vast range of fruits with a different composition of nutrients and bioactive compounds (Rodrigo and Zacarías, 2006; Cano et al., 2008; Ma et al., 2020a). Current information about tocopherol contents in *Citrus* fruit is very limited. Tocopherols accumulate in the flavedo (external colored part of the peel) of mandarin, orange, lemon, grapefruit, and other less known species in the range of 65–130 μg g^–1^, and mainly in the forms of α- and γ-tocopherol, with composition varying according to the specie (Assefa et al., 2017; Rey et al., 2021a, b). In the pulp, tocopherols have also been detected in fruit of mandarin, grapefruit, and orange in the form of α-tocopherol and at concentrations lower than in the flavedo (1.6–25 μg g^–1^) (Chun et al., 2006; Cardeñosa et al., 2015). Recently, genes involved in the tocopherol-core pathway as well as genes regulating the supply of the precursors HGA and PPP have been identified in the *Citrus* genome. Analysis of their transcriptional profiling in the flavedo of mandarins and grapefruits in response to cold stress suggested candidate genes and mechanisms regulating tocopherol accumulation in each specie (Rey et al., 2021a, b). However, the temporal changes of tocopherols and the regulation of their synthesis during maturation of *Citrus* fruit have not been explored yet. Therefore, the aim of the present work was to perform a comparative study of tocopherol accumulation during on-tree fruit maturation in different *Citrus* genotypes belonging to the main horticultural *Citrus* groups: orange, mandarin, lemon, and grapefruit. The relationship between tocopherol accumulation and the expression of tocopherol-biosynthetic genes in the flavedo and pulp of fruit from the selected species and ripening stages was also investigated.

## Materials and Methods

### Plant Material

Fruit of four different genotypes belonging to the main horticultural groups of *Citrus* species: grapefruit (*Citrus paradisi* Macfad.) cv. ‘Marsh’, lemon [*Citrus limon* (L.) Burm. F] cv. ‘Fino’, sweet orange [*Citrus sinensis* (L.) Osbeck] cv. ‘Washington Navel’, and mandarin (*Citrus clementina* Hort. ex Tanaka) cv. ‘Clemenules’, were selected for this study. Trees were located in the Citrus Germplasm Bank at the Instituto Valenciano de Investigaciones Agrarias (IVIA) located in Moncada (Valencia, Spain; 39°35′22″N, 0°23′40″W; 55 m elevation above the sea level), and cultivated under standard agronomical conditions. Fruits were harvested from adult trees (more than 10 years old) grafted on Carrizo citrange rootstock [*C. sinensis* (L.) Osb. × *Poncirus trifoliata* (L.) Raf.], growing in a loamy-sand soil with drip irrigation. Fertilization, pruning, pest management, and other cultural conditions were performed according to the protocol established by IVIA for the Citrus Germplasm Bank. Fruits were harvested at four maturation stages: immature green (IG), mature green (MG), breaker (Br), and mature fruit (M), and the specific harvest dates for each genotype are detailed in Supplementary Table 1. For each genotype and sampling date three biological replicates of 10–15 fruits, collected randomly from the outside of the tree canopy of 2–3 trees, were used. Fruits were delivered to the laboratory and selected for color uniformity and free of any peel defect or damage, and the flavedo (external colored layer of fruit peel) and pulp (juice vesicles) were excised with a scalpel, frozen in liquid nitrogen and ground to a fine powder using an electric grinder with liquid nitrogen. Samples were stored at −80°C until analysis.

### Tocopherol Extraction and Quantification

Tocopherol extraction of the flavedo and pulp, and quantification by HPLC coupled to fluorescence detector, was carried out following the procedure described in Rey et al. (2021a). In summary, flavedo and pulp material (200 and 500 mg, respectively) were extracted with methanol, Tris buffer (50 mM Tris pH 7.5) with 1 M NaCl, and dichloromethane in a mortar and pestle with sea sand as an abrasive. After vortex-mixing, samples were sonicated for 5 min and centrifuged for 10 min at 3000 × *g* and 4°C. The dichloromethane phase was recovered in a glass tube, and the methanol phase was re-extracted with dichloromethane. The pooled dichloromethane extracts were then dried under nitrogen gas and stored at −20°C until HPLC analysis. For quantification, dried extracts were re-suspended in ethyl acetate (500–700 μl) and a dilution (1:15 for flavedo extracts and 1:2 for pulps) was carried out. For analysis, 20 μl of the diluted extract were injected in a Waters HPLC system (Acquity^®^ Arc^TM^, Waters) coupled with a fluorescence detector (2475 FLR Detector, Waters). Tocopherol separation was done using a C30 column, 150 mm × 4.6 mm, 3 μm (YMC, Teknokroma, Spain), and a ternary gradient elution with methanol, water, and methyl *tert*-butyl ether at 1 ml min^–1^ flow (Rey et al., 2021a). Elution of tocopherols was monitored by fluorescence at an excitation wavelength of 296 nm and emission wavelength of 340 nm. Identification and quantification of the different tocopherols was achieved by comparison with the retention times and calibration curves for tocopherol standards (Sigma-Aldrich). All procedures were carried out on ice and under dim light to prevent photo-degradation. Total tocopherol content was calculated as the sum of the tocopherol isoforms, and concentrations are expressed as μg g of fresh weight (FW). Results are the mean of two biological replicates (mean ± SD).

### RNA Extraction and cDNA Synthesis

Extraction of total RNA was different according to the fruit tissue. Total RNA was isolated from flavedo tissue using the RNeasy Plant Mini Kit (Qiagen), while the extraction from the pulp was done using the protocol described in Rodrigo et al. (2004). Once total RNA was isolated, DNA traces were removed by treating RNA with DNase I (DNA free, DNase treatment and removal, and Ambion) following the manufacturer’s instructions. RNA concentration was later quantified by spectrophotometric analysis (NanoDrop, Thermo Fisher Scientific) and RNA quality was verified by agarose-gel electrophoresis with GoodView^®^ Nucleic Acid Stain (SBS Genetech). For cDNA synthesis, 5 μg of total RNA were reverse-transcribed using the SuperScript III Reverse Transcriptase (Invitrogen) in a final volume of 20 μl, following the manufacturer’s procedure.

### Gene Expression Analysis by Quantitative Real-Time PCR

Gene expression was evaluated by quantitative real-time PCR in a LightCycler 480 instrument (Roche), using the LightCycler 480 SYBRGreen I Master kit (Roche) and following the manufacturer’s instructions. Previously published oligonucleotides primers were used for the amplification of the following *C. sinensis* genes related to tocopherol synthesis: *DXS1*, *DXS2*, *GGPPS1*, *GGPPS6*, *GGDR*, *VTE5*, *VTE6*, *TAT1*, *HPPD*, *VTE2*, *VTE3a*, *VTE3b*, *VTE1*, and *VTE4* (Rey et al., 2021a). The primers sequences used for RT-qPCR analyses is listed in Supplementary Table 2. For all the genes analyzed, RT-qPCR conditions consisted of an initial pre-incubation at 95°C for 10 min, followed by 40 cycles of 10 s at 95°C for denaturation, 10 s at 59°C for annealing, and 10 s at 72°C for extension. For each amplification reaction 1 ml of 10 times diluted first-strand cDNA, containing approximately 100 ng of cDNA, was used. Specificity of the PCR reaction in the different *Citrus* species was assessed by the melting point analysis (Tm) after the amplification steps. For expression measurements, we used the LightCycler 480 Software release 1.5.0, version 1.5.0.39 (Roche) and calculated relative expression levels using the Relative Expression Software Tool (REST) (Pfaffl et al., 2002) with an external reference sample. The reference sample consisted of flavedo pool of a mix of cDNA from immature to mature fruit of different *Citrus* species and cultivars, including those used in this work. The expression value obtained for each gene in the reference sample was set to 1, and it was used for the calculation of relative gene expression in all the flavedo and pulp samples analyzed in this work. Normalization was performed using *ACTIN* as a housekeeping gene (Alós et al., 2014; Zacarías-García et al., 2021). Results are the mean of three biological replicates (mean ± SE).

### Correlation Matrix and Networks

To gain insight into possible common candidate genes regulating tocopherol accumulation in *Citrus* fruit, a correlation matrix and network was built independently for the flavedo and pulp, based on procedures described by Diretto et al. (2010). First, a Pearson’s correlation analysis was carried out for each fruit tissue, taking into account all the metabolite (tocopherol contents) and expression data (relative gene expression levels). For each tissue, the input data for the calculation of correlation coefficients was the fold-change (log2 transformed) relative to the IG stage of grapefruit for all the variables, species, and maturation stages. The results were displayed as a matrix, in which each circle represents the correlation between the gene/metabolite in the column heading and the gene/metabolite in the row heading, with their size and color intensity being proportional to the absolute correlation coefficient. Correlation coefficients values are summarized in Supplementary Tables 7, 8, where significant correlations are marked with an asterisk (*p*-value ≤ 0.05). Correlation analysis were carried in RStudio (version 1.3.1093, RStudio Team, PBC, Boston, MA, United States) using the function “*cormat*” and visualized using the function “*corrplot*” of the package “*ggplot2*.” Subsequently, each correlation matrix was transformed into a correlation network to highlight the different connections between tocopherol contents and gene expression in the different tissues, and also possible co-regulation among genes. For the construction of the correlation networks only significant correlations were taken into account (*p*-value ≤ 0.05), and the networks were assembled manually using Cytoscape version 3.8.2 (National Institute of General Medical Sciences, Bethesda, MD, United States). The correlation network was displayed in a graph illustrating all-versus-all correlations (only significant) among the variables analyzed and it is composed of nodes, which represented tocopherol contents and gene expression levels, and edge lines which represented the links between those nodes. Positive correlations are represented in red and negative ones in blue, and color intensity is proportional to the absolute correlation coefficient.

### Statistical Analyses

Tocopherol contents and relative gene expression data were subjected to a one-way analysis of variance (ANOVA) followed by the multiple comparison Tukey’s test (significance level at *p* ≤ 0.05), to determine significant mean differences. This analysis was carried to assessed differences among maturation stages for each specie, and additionally the same analysis was done to assess differences among species for a specific maturation stage. The InfoStat software (version 2018, Grupo InfoStat, Córdoba, Argentina) was used for the statistical analyses.

## Results

### Changes in Tocopherol Content During Fruit Maturation of Four *Citrus* Species: Grapefruit (*Citrus paradisi*), Lemon (*Citrus limon*), Sweet Orange (*Citrus sinensis*), and Mandarin (*Citrus clementine*) During Ripening

**FIGURE 2:**
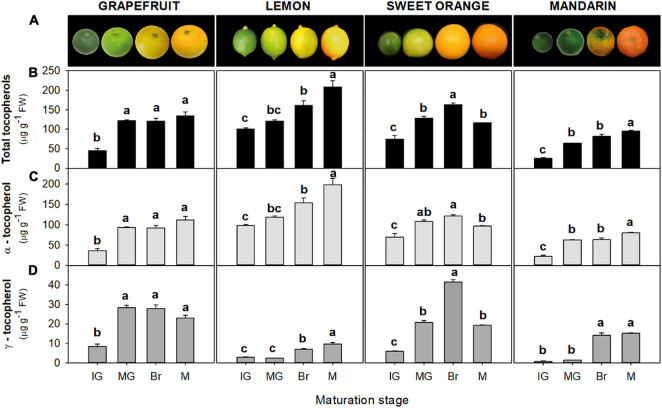
Fruit external appearance **(A)** and total tocopherol **(B)**, α-tocopherol **(C)**, and γ-tocopherol content **(D)** in the flavedo during fruit maturation of grapefruit (*C. paradisi*), lemon (*C. limon*), sweet orange (*C. sinensis*), and mandarin (*C. clementine*). Contents are expressed as μg g^– 1^ of fresh weight. The data are mean ± SE of at least two replicates (Supplementary Table 9). Maturation stages correspond to immature green (IG), mature green (MG), breaker (Br), and mature (M). Different letters indicate significant mean differences among maturation stages for each fruit specie (Tukey’s test, *p* ≤ 0.05).

**FIGURE 3:**
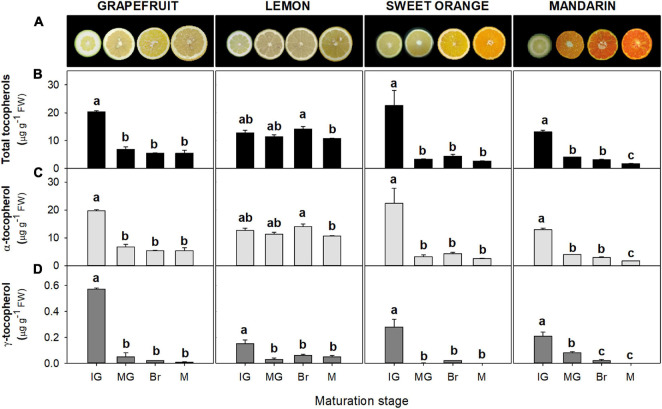
Fruit internal appearance **(A)** and total tocopherol **(B)**, α-tocopherol **(C)**, and γ-tocopherol content **(D)** in the pulp during fruit maturation of grapefruit (*C. paradisi*), lemon (*C. limon*), sweet orange (*C. sinensis*), and mandarin (*C. clementine*). Contents are expressed as μg g^– 1^ of fresh weight. The data are mean ± SE of at least two replicates (Supplementary Table 10). Maturation stages correspond to immature green (IG), mature green (MG), breaker (Br), and mature (M). Different letters indicate significant mean differences among maturation stages for each fruit specie (Tukey’s test, *p* ≤ 0.05).

Tocopherols were detected in the flavedo and pulp of the four *Citrus* genotypes selected at the four successive stages of fruit maturation (Figures 2, 3). The isoforms α- and γ-tocopherol were identified in all samples, while the presence of δ-tocopherol was only detected in a few samples at concentrations below 10 and 6 ng g^–1^ FW in the flavedo and pulp, respectively. α-Tocopherol was the predominant form in all species and stages, accounting on average for 85% of total tocopherols in the flavedo and 99% in the pulp. In the flavedo of all species, tocopherol content was higher than in the pulp and contents gradually increased with maturation (Figure 2). By contrast, total tocopherols in the pulp decreased sharply after the IG stage and, at the mature (M) stage, the content was between 3 and 8-times lower than in IG, with the exception of lemon where levels remained nearly constant during ripening (Figure 3). As a result, differences between tissues became greater during maturation, with contents being 2–7 times higher in the flavedo than in the pulp at the IG stage and more than 20–50 times higher at full maturity (M) (Figures 2, 3).

Differences in tocopherol content in the flavedo and pulp were observed among genotypes (Figures 2, 3 and Supplementary Tables 3, 4). At the IG stage, total tocopherol contents (as the sum of α- and γ-tocopherol) were significantly higher in the flavedo of lemon (∼101 μg g^–1^ FW), followed by sweet orange (∼75 μg g^–1^ FW), and almost 2- and 4-times lower in the flavedo of grapefruit (∼45 μg g^–1^ FW) and mandarin (∼23 μg g^–1^ FW), respectively (Figure 2 and Supplementary Table 3). During maturation, total tocopherols increased in the four species, but the magnitude of the increase varied among species (Figure 2). At the M stage, higher concentrations of tocopherols were detected in the flavedo of lemon (∼208 μg g^–1^ FW) than in grapefruit (∼134 μg g^–1^ FW), sweet orange (∼116 μg g^–1^ FW), and mandarin (∼95 μg g^–1^ FW) (Supplementary Table 3). In contrast to the other species, contents in sweet orange increased to a maximum at the breaker (Br) stage (∼160 μg g^–1^ FW) and then decreased toward M fruit (Figure 2B). Contents of α-tocopherol reflected the differences among species described for total tocopherol. In IG fruit, α-tocopherol levels ranged from 22 to 98 μg g^–1^ FW and increased to levels between 80 and 199 μg g^–1^ FW at the M stage, with lemon fruit accumulating the highest content (Figure 2C and Supplementary Table 3). γ-Tocopherol ranged from 0.75 to 8.4 μg g^–1^ FW at the IG stage, with lemon and mandarin accumulating the lowest content (<3 μg) (Figure 2D and Supplementary Table 3). This tocopherol isoform increased during maturation in the flavedo of all species, reaching maximum values at the Br or M stages, between 10 μg g^–1^ in lemon and 42 μg g^–1^ in sweet orange (Figure 2D). It should be noticed that in all the ripening stages, the flavedo of lemon and mandarin showed lower concentrations of γ-tocopherol in comparison to sweet orange and grapefruit (Figure 2D and Supplementary Table 3).

The pulp of sweet orange, mandarin, and grapefruit showed the highest levels of total tocopherol at the IG stage (∼13–23 μg g^–1^ FW), which decreased at the MG stage (more than fourfold) (Figure 3). In lemon, changes during ripening were minor and levels remained almost constant (∼11–14 μg g^–1^ FW). In M fruit, the pulp of lemon accumulated the highest total contents, followed by grapefruit and lastly orange and mandarin (Supplementary Table 4). As in the flavedo, α-tocopherol contents reflected the main differences in total tocopherols among species and during maturation (Figure 3C and Supplementary Table 4). Contents in the pulp ranged from 13 to 22 μg g^–1^ FW at IG stage, and 2 to 11 μg g^–1^ FW at M fruit. The levels of γ-tocopherol in the pulp of the four species were below 1 μg g^–1^ FW at IG stage and decreased during maturation in the four species (Figure 3D).

### Expression Profile of Genes Involved in Tocopherol Synthesis in the Flavedo During Fruit Maturation of Four *Citrus* Species: Grapefruit (*Citrus paradisi*), Lemon (*Citrus limon*), Sweet Orange (*Citrus. sinensis*), and Mandarin (*Citrus clementine*)

**FIGURE 4:**
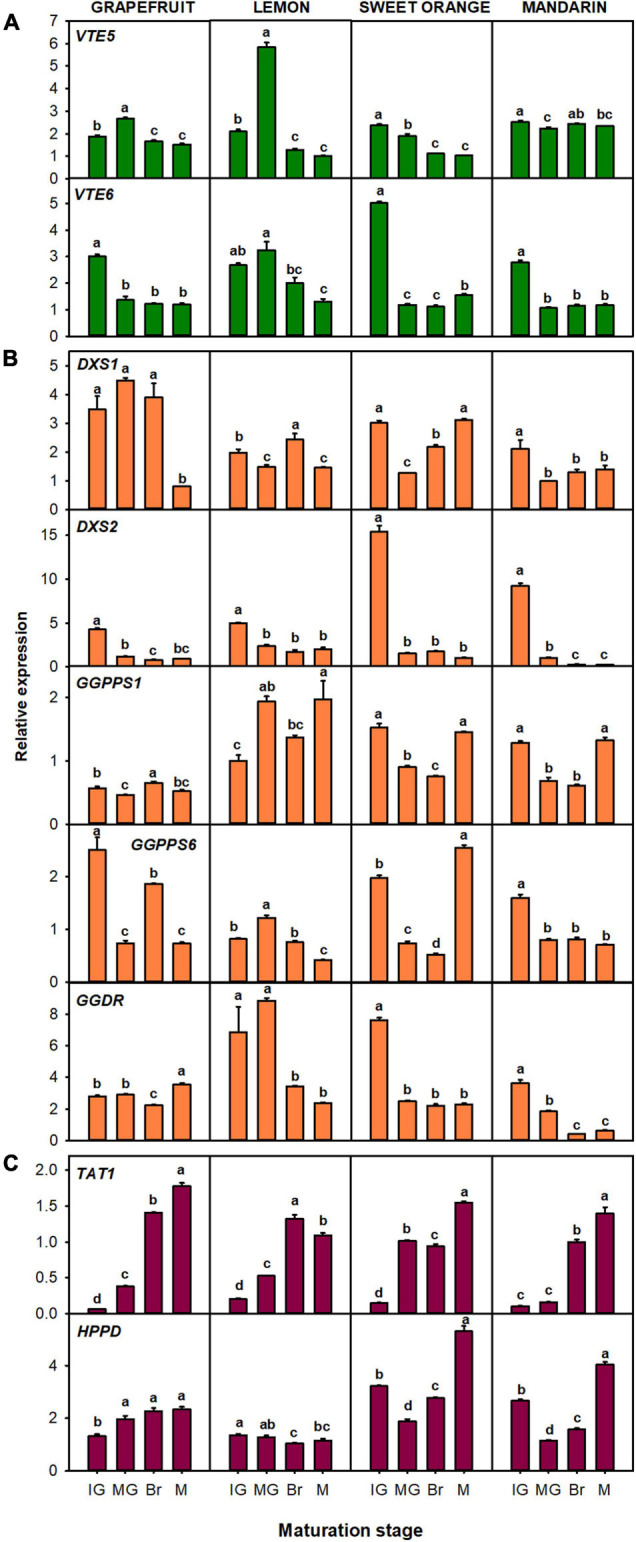
Relative expression of genes involved in the synthesis of precursor PPP (phytyl pyrophosphate), through the recycling of free phytol **(A)** and MEP pathway **(B)**, and of precursor HGA (homogentisate) through the SK pathway **(C)**, in the flavedo during fruit maturation of grapefruit (*C. paradisi*), lemon (*C. limon*), sweet orange (*C. sinensis*), and mandarin (*C. clementine*). The genes analyzed were *VTE5* (phytol kinase), *VTE6* (phytyl-P kinase), *DXS1* and *DXS2* (1-deoxy-D-xylulose-5-phosphate synthase 1 and 2), *GGPPS1* and *GGPPS6* (geranylgeranyl pyrophosphate synthase 1 and 6), *GGDR* (geranylgeranyl diphosphate reductase), *TAT1* (tyrosine aminotransferase), and *HPPD* (4-hydroxyphenylpyruvate dioxygenase). Maturation stages correspond to immature green (IG), mature green (MG), breaker (Br), and mature (M). The data are mean ± SE of at least three replicates (Supplementary Table 11). Different letters indicate significant mean differences among maturation stages for each fruit specie (Tukey’s test, *p* ≤ 0.05).

**FIGURE 5:**
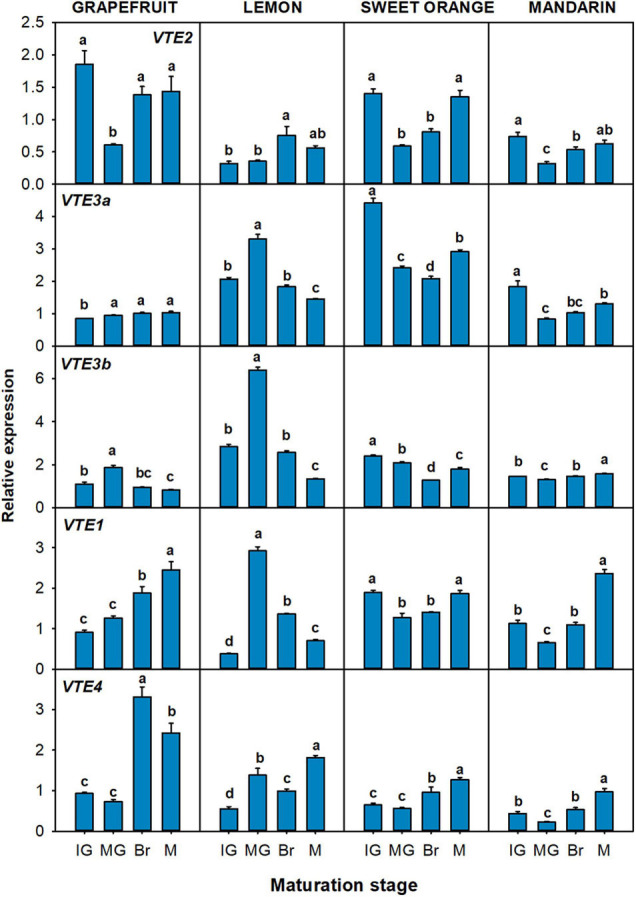
Relative expression of genes of the tocopherol-core pathway in the flavedo during fruit maturation of grapefruit (*C. paradisi*), lemon (*C. limon*), sweet orange (*C. sinensis*), and mandarin (*C. clementine*). The genes analyzed were *VTE2* (HPT, homogentisate phytyl transferase), *VTE3a* and *VTE3b* (MPBQ-MT, 2-methyl-6-phytyl-1,4-benzoquinol methyltransferase a and b), *VTE1* (TC, tocopherol cyclase), and *VTE4* (γ-TMT, γ-tocopherol methyltransferase). Maturation stages correspond to immature green (IG), mature green (MG), breaker (Br), and mature (M). The data are mean ± SE of at least three replicates (Supplementary Table 11). Different letters indicate significant mean differences among maturation stages for each fruit specie (Tukey’s test, *p* ≤ 0.05).

The relative expression of genes related to tocopherol precursors production: *VTE5*, *VTE6*, *DXS1*, *DXS2*, *GGPPS1*, *GGPPS6*, *GGDR*, *TAT1*, and *HPPD* (Figure 4), and to the tocopherol-core pathway: *VTE2*, *VTE3a*, *VTE3b*, *VTE1*, and *VTE4* (Figure 5) were evaluated in the flavedo of the four selected *Citrus* species during fruit maturation. Changes in the expression of the genes involved in the synthesis of the precursor PPP, which includes genes involved in chlorophyll degradation (Figures 1, 4A) and of the MEP pathway (Figures 1, 4B) during fruit ripening, were in general gene- and specie-dependent, but tended to be down-regulated. Of the genes involved in the recycling of free phytol to form PPP (Figure 4A), a significant down-regulation of *VTE6* during fruit ripening (two- to threefold decrease) was detected in fruits of the four species. *VTE5* expression also decreased in the four species, although with a transient induction at the MG stage in lemon and grapefruit. Regarding genes of the MEP pathway (Figure 4B), a decrease in the expression of *DXS2* was detected during fruit ripening in the four species, and of *GGDR* in lemon, orange, and mandarin. In the flavedo of grapefruit, the expression decreased at Br stage but increased again at the M stage. No clear common trend among the four species was found in the expression of *DXS1* and the two *GGPPS* paralogous during fruit maturation, although *DXS1* and *GGPPS6* tended to decrease in most species (Figure 4B). By contrast, the genes involved in the synthesis of the precursor HGA, *TAT1* and *HPPD*, were in general induced during maturation (Figure 4C). The expression of *TAT1* was significantly up-regulated in the four species, showing levels 6- to 28-fold higher at Br and M stages than at IG. The relative expression of *HPPD* gradually increased in grapefruit and at the last stage of maturation in sweet orange and mandarin, but decreased in lemon (Figure 4C).

Expression of the tocopherol-core pathway genes during maturation varied among species, but most of the genes were induced or maintained during ripening (Figure 5). The gene *VTE2*, which controls the condensation of PPP with HGA, was induced almost two-times in lemon at the Br stage, while it displayed a transient but significant down-regulation between the MG and Br stage in the other three genotypes. The two *VTE3* isoforms were significantly down-regulated in lemon and orange, although a transient increase at the MG stage was detected in lemon. In grapefruit *VTE3a* increased after the IG stage, while it decreased in mandarin’s flavedo after the same stage. On the other hand, expression of *VTE3b* decreased in grapefruit, although with a peak at the MG stage, whereas it was induced at the M stage in mandarin fruit. The gene *VTE1* displayed an induction in grapefruit and mandarin (twofold increase), while its expression was maintained with transient variations at the MG and Br stage in sweet orange. In lemon, *VTE1* showed a similar pattern of expression to the *VTE3* isoforms, with a sharp and induction at the MG stage that decreased at later stages. Finally, *VTE4* displayed the most consistent expression pattern among the four species, with a significant up-regulation (two and three-times) during maturation.

### Expression Profile of Genes Involved in Tocopherol Synthesis in the Pulp During Fruit Maturation of Four *Citrus* Species: Grapefruit (*Citrus paradisi*), Lemon (*Citrus limon*), Sweet Orange (*Citrus sinensis*), and Mandarin (*Citrus clementine*)

**FIGURE 6:**
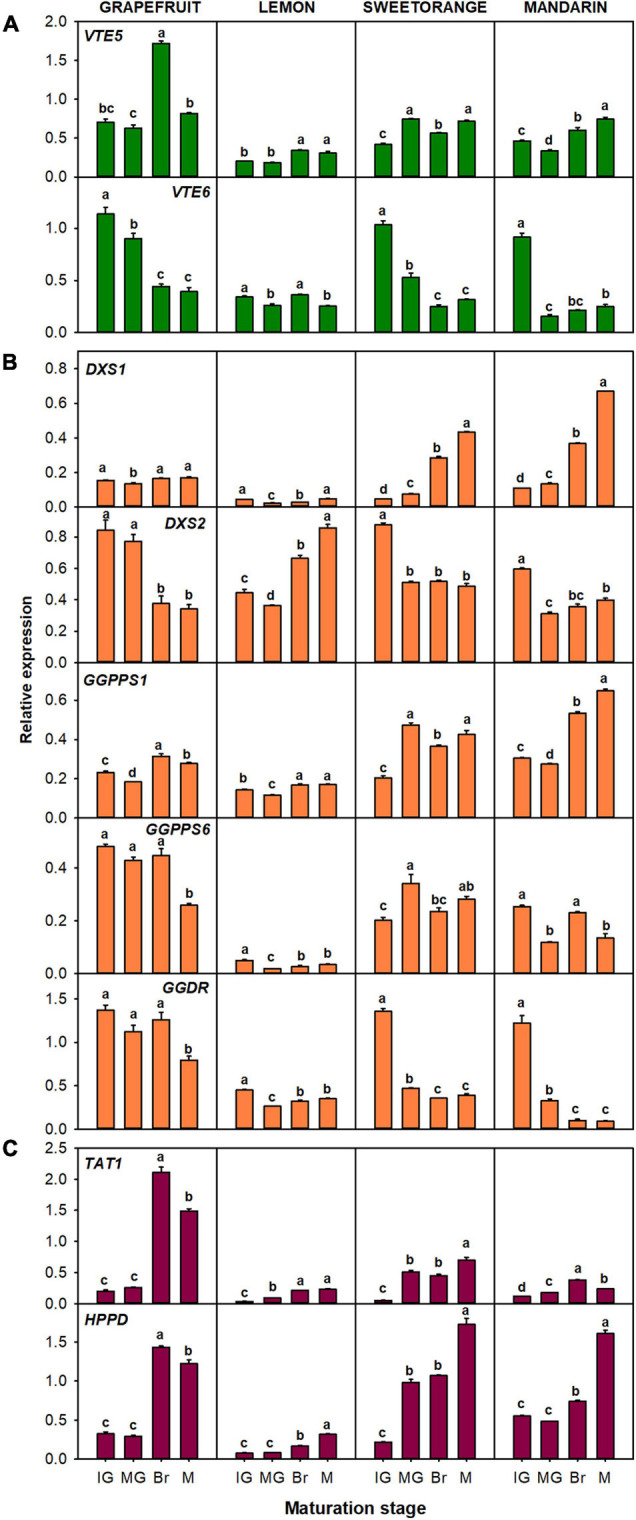
Relative expression of genes involved in the synthesis of precursor PPP (phytyl pyrophosphate), through the recycling of free phytol **(A)** and MEP pathway **(B)**, and of precursor HGA (homogentisate) through the SK pathway **(C)**, in the pulp during fruit maturation of grapefruit (*C. paradisi*), lemon (*C. limon*), sweet orange (*C. sinensis*), and mandarin (*C. clementine*). The genes analyzed were *VTE5* (phytol kinase), *VTE6* (phytyl-P kinase), *DXS1* and *DXS2* (1-deoxy-D-xylulose-5-phosphate synthase 1 and 2), *GGPPS1* and *GGPPS6* (geranylgeranyl pyrophosphate synthase 1 and 6), *GGDR* (geranylgeranyl diphosphate reductase), *TAT1* (tyrosine aminotransferase), and *HPPD* (4-hydroxyphenylpyruvate dioxygenase). Maturation stages correspond to immature green (IG), mature green (MG), breaker (Br), and mature (M). The data are mean ± SE of at least three replicates (Supplementary Table 12). Different letters indicate significant mean differences among maturation stages for each fruit specie (Tukey’s test, *p* ≤ 0.05).

**FIGURE 7:**
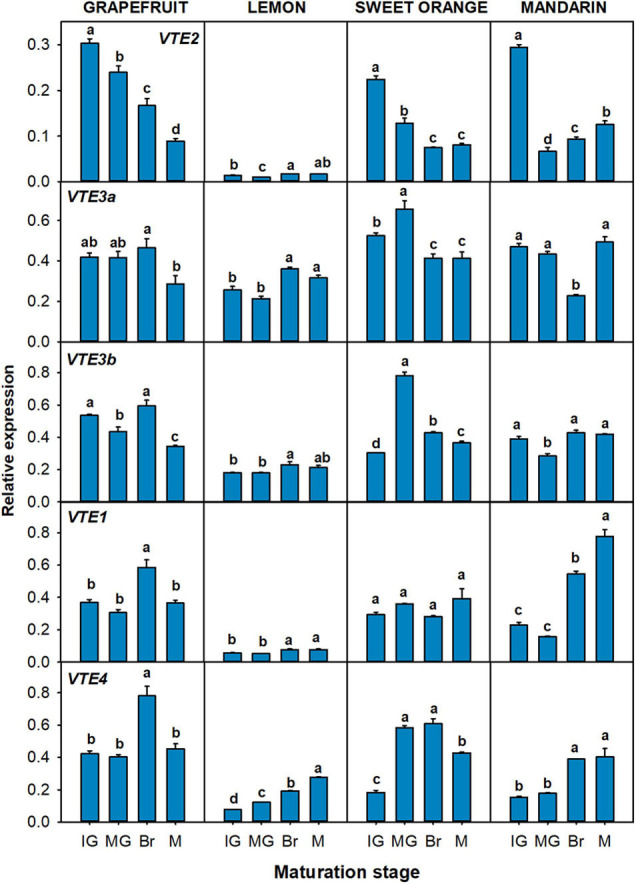
Relative expression of genes of the tocopherol-core pathway in the pulp during fruit maturation of grapefruit (*C. paradisi*), lemon (*C. limon*), sweet orange (*C. sinensis*), and mandarin (*C. clementine*). The genes analyzed were *VTE2* (HPT, homogentisate phytyl transferase), *VTE3a* and *VTE3b* (MPBQ-MT, 2-methyl-6-phytyl-1,4-benzoquinol methyltransferase a and b), *VTE1* (TC, tocopherol cyclase), and *VTE4* (γ-TMT, γ-tocopherol methyltransferase). Maturation stages correspond to immature green (IG), mature green (MG), breaker (Br), and mature (M). The data are mean ± SE of at least three replicates (Supplementary Table 12). Different letters indicate significant mean differences among maturation stages for each fruit specie (Tukey’s test, *p* ≤ 0.05).

The relative expression of the genes related to the production of tocopherol precursors: *VTE5*, *VTE6*, *DXS1*, *DXS2*, *GGPPS1*, *GGPPS6*, *GGDR*, *TAT1*, and *HPPD* (Figure 6), and to the tocopherol-core pathway: *VTE2*, *VTE3a*, *VTE3b*, *VTE1*, and *VTE4* (Figure 7), were analyzed in the pulp of fruit from the selected genotypes. In general, temporal changes in the expression of genes involved in PPP synthesis in the pulp were dependent on the gene and specie, but similarities for certain genes were found. Concerning genes of chlorophyll degradation, *VTE5* transcripts were significantly up-regulated in lemon, orange, and mandarin during ripening and only transiently at the Br stage in grapefruit, while the expression of *VTE6* was significantly down-regulated in the pulp of the four species (Figure 6A). Of the MEP pathway genes, expression of *DXS1* was induced in sweet orange and mandarin (more than twofold) but remained relatively unaltered in grapefruit and lemon (only with transient decreases mid-ripening) (Figure 6B). Interestingly, expression of *DXS2* was induced in lemon but down-regulated in the pulp of the other *Citrus* species. Furthermore, while *GGPPS1* was significantly induced in the four species, the isoform *GGPPS6* was only induced in orange and down-regulated in the other species. The expression of *GGDR* decreased during maturation in the four species, but the reduction was more marked in sweet orange and mandarin than in the other species. On the other hand, the expression of both *TAT1* and *HPPD* genes, involved in HGA synthesis, was up-regulated during ripening in the pulp of the four citrus fruits.

In relation with the genes of the tocopherol-core pathway, no common expression trend among genotypes was detected (Figure 7). The gene *VTE2* was significantly down-regulated during fruit maturation in grapefruit, sweet orange, and mandarin, whereas no difference between the M and IG stages was detected in lemon. Moreover, the gene *VTE4* was significantly up-regulated during maturation in lemon, orange, and mandarin, but only transitionally at Br stage in grapefruit. In relation to the other genes, expression of *VTE3a* was induced in lemon while it remained almost unaltered in grapefruit and mandarin (with transient significant changes at the Br stage). A different pattern for the *VTE3b* isoform was detected in grapefruit, lemon, and mandarin, with its expression being down-regulated in grapefruit and relative constant in lemon and mandarin. In orange, both isoforms displayed the same expression pattern, with a significant increase at the MG stage that later decreased again. Expression of *VTE1* showed minor alterations in the pulp of grapefruit and sweet orange, but was significantly induced in lemon and mandarin.

### Correlation and Network Analysis of Tocopherol Contents and Relative Expression of the Genes Involved in Tocopherol Synthesis in the Flavedo and Pulp During Fruit Maturation of Four *Citrus* Species: Grapefruit (*Citrus paradisi*), Lemon (*Citrus limon*), Sweet Orange (*Citrus sinensis*), and Mandarin (*Citrus clementine*)

**FIGURE 8:**
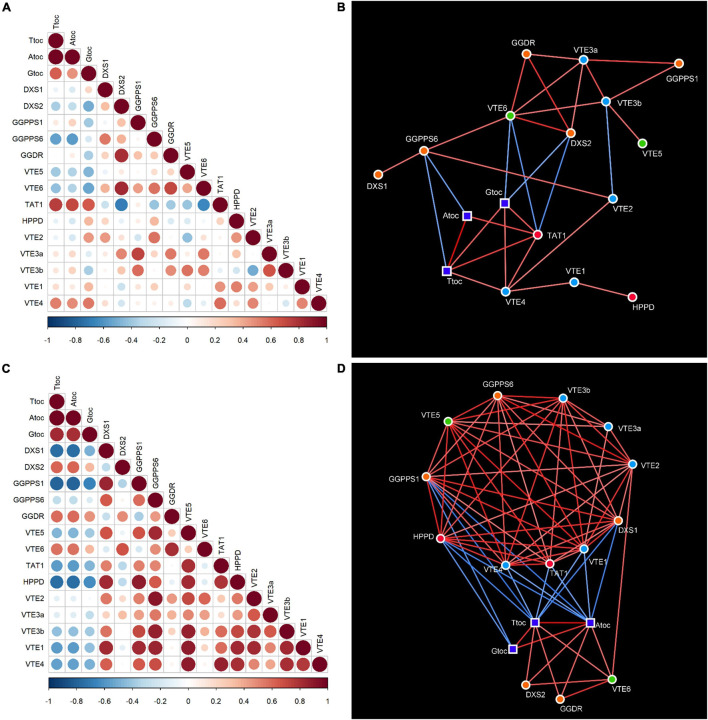
Correlation matrices **(A,C)** and networks **(B,D)** of tocopherol contents and expression of genes involved in tocopherol synthesis in the flavedo **(A,B)** and pulp **(C,D)** during fruit maturation of grapefruit (*C. paradisi*), lemon (*C. limon*), sweet orange (*C. sinensis*), and mandarin (*C. clementine*). Positive and negative correlations in the matrices are shown in different shades of red and blue, respectively, with the size of the circle and color intensity indicating the magnitude of Pearson’s correlation coefficient. In the correlation networks, square nodes represent tocopherol contents (Ttoc, total tocopherols; Atoc, α-tocopherol; Gtoc, γ-tocopherol) and circle nodes represent genes (green, genes involved in the recycling of phytol; orange, genes of the MEP pathway; red, genes of the SK pathway; and blue, genes of the tocopherol-core pathway). Lines joining the nodes represent correlations (edges); positive correlations are shown in red, while negative correlations are in blue, and the color intensity represents the strength of the correlation (absolute value of the Pearson’s correlation coefficient). Only significant correlations (*p*-value ≤ 0.05) were taken into account for constructing the correlation networks. Values for the correlation coefficients and significances are detailed in Supplementary Tables 7, 8, 13, 14.

To better understand the relationship between gene expression and the accumulation of tocopherols, a correlation matrix (Figures 8A,C) and network analysis (Figures 8B,D) were built independently for the flavedo and pulp, using data of the four species at the four maturation stages. The network analysis, in which only significant correlations were taken into account, revealed that tocopherols (total, α- and γ-forms) and all genes analyzed were present in both the flavedo and pulp networks, arranged in an interconnected group in each tissue (Figure 8). However, the number of interconnections in the networks was different between tissues, with a media of 3.76 edges in the flavedo and 8.82 in the pulp, and the number of negative links between metabolite nodes and the expression of genes was higher in the pulp than in the flavedo (Figures 8B,D).

In the flavedo, *VTE6* and *TAT1* were the most interconnected genes (6–7 edges), but *GGPPS6*, *VTE3a*, *VTE3b*, and *VTE4* also had links above the median (Figure 8B). Not surprisingly, α-tocopherol and γ-tocopherol showed a strong and positive relationship with total tocopherols, but the correlation between α- and γ-tocopherol was not significant (Figures 8A,B). Moreover, tocopherols were positively correlated with the genes *TAT1*, of the SK pathway, and *VTE4*, of the tocopherol core pathway, suggesting that both *TAT1* and *VTE4* are key genes modulating tocopherol accumulation in the flavedo (Figures 8A,B). On the other hand, total tocopherols and α-tocopherol were negatively correlated with the gene *GGPPS6* of the MEP pathway, while γ-tocopherol displayed a negative correlation with the genes *VTE6* and *DXS2* (Figures 8A,B). Interestingly, the gene *VTE6* seemed highly connected to genes of the MEP pathway (*DXS2*, *GGDR*, and *GGPPS6*) and with both isoforms of *VTE3* (Figure 8B), suggesting that they are co-regulated in the flavedo during ripening.

In the pulp, 10 genes: *DXS1*, *GGPPS1*, *GGPPS6*, *VTE5*, *TAT1*, *HPPD*, *VTE2*, *VTE3b*, *VTE1*, and *VTE4*, showed a number of links above the median, being *GGPPS1*, *HPPD*, *VTE1*, *DXS1*, and *VTE4* the most interconnected genes (11–12 edges) (Figures 8C,D). A strong positive correlation was detected between α- and γ-tocopherol in the pulp (Figures 8C,D). The network analysis in the pulp also revealed a positive correlation of total and α-tocopherol with the genes *VTE6*, *DXS2*, and *GGDR* (Figures 8C,D). Tocopherols were negatively correlated with *GGPPS1* and *HPPD* and, in the case of total tocopherols and α-tocopherol, also with *DXS1*, *TAT1*, *VTE1*, and *VTE4* (Figures 8C,D). Similar to what was observed in the flavedo, a connection between *VTE6* and the genes *DXS2* and *GGDR* of the MEP pathway was detected. Furthermore, other genes were positively co-regulated in the pulp, including genes of the MEP pathway (*GGPPS6*, *GGPPS1*, and *DXS1*), involved in phytol recycling (*VTE5*), of the SK pathway (*HPPD* and *TAT1*) and of the tocopherol-core pathway (*VTE2*, *VTE3b*, *VTE1*, and *VTE4*).

## Discussion

Taking advantage of the genetic and phenotypical diversity of the genus *Citrus*, the aim of this work was to investigate the changes in tocopherol contents and their regulation during maturation of fruit of four *Citrus* species belonging to the main horticultural groups: grapefruit, lemon, sweet orange, and mandarin. Tocopherols were identified in all the selected *Citrus* species throughout maturation, with contents varying between tissues, maturation stages, and genotypes (Figures 2, 3 and Supplementary Tables 3, 4). The tocopherol profile was in agreement with previous reports, with α- and γ-tocopherol being the main forms detected in *Citrus* fruit (Assefa et al., 2017; Rey et al., 2021a, b). However, predominance of one form or another seems to be dependent on the *Citrus* specie, as similar or higher γ-tocopherol contents have been detected in less common Korean *Citrus* genotypes (Assefa et al., 2017). In fruit of other species, the prevalence of α- or γ-tocopherol is also specie-specific and, while α-tocopherol is the main form in tomato, pepper, mango, grape, olive, and avocado (Koch et al., 2002; Horvath et al., 2006b; Quadrana et al., 2013; Singh et al., 2017; Georgiadou et al., 2019; Vincent et al., 2020), γ-tocopherol accumulates at higher concentrations in zucchini and raspberry fruit (Carvalho et al., 2013; Rodov et al., 2020). In our experimental conditions, β-tocopherol was not detected, and δ-tocopherol was only identified in some samples but at levels below the limit of quantification, indicating that the δ-/β-tocopherol branch may not have a significant contribution to the tocopherol pool in citrus fruit.

Many physiological and metabolic processes are independently regulated in the flavedo and pulp of *Citrus* fruit (Tadeo et al., 2020), and this seems to be the case for tocopherol metabolism as contents were higher in the flavedo than the pulp (Figures 2, 3). Similarly, higher concentrations of other bioactive compounds have been reported in the flavedo than in the pulp of citrus (Alquézar et al., 2008, 2013; Alós et al., 2014; Assefa et al., 2017). A possible explanation for this is that the direct exposure of the flavedo to environmental stresses could lead to a higher demand for antioxidants to cope with these adverse conditions. In relation to this, light could play a relevant role either as a stress factor or by its direct impact in the regulation of tocopherol biosynthesis. Tocopherols are involved in the photo-protection of plants (Spicher et al., 2017; Ma et al., 2020b), and increases in contents have been reported in response to high light stress (Collakova and DellaPenna, 2003b; Havaux et al., 2005). Furthermore, a positive role of light in the transcriptional regulation of certain tocopherol biosynthetic genes has been proposed in grapefruit (Rey et al., 2021b) and tomato (Gramegna et al., 2019).

The differences detected between fruit tissues were not only quantitative but also in the pattern of accumulation during maturation (Figures 2, 3), suggesting that different mechanisms may operate in the regulation of tocopherol synthesis in the flavedo and pulp of *Citrus* fruit. The differential accumulation of tocopherols between citrus fruit tissues may also be related to their chlorophyll contents, as an alternative source of PPP is through the recycling of free phytol formed during the degradation of chlorophylls (Figure 1; Valentin et al., 2006; Georgiadou et al., 2015; vom Dorp et al., 2015; Almeida et al., 2016). While chlorophyll content in the pulp of *Citrus* fruit is negligible or only detected in IG fruit, concentrations in the flavedo are high throughout development and decrease notably with the transition of chloroplasts to chromoplasts (Alquézar et al., 2008, 2013; Lado et al., 2015, 2019). Therefore, the higher concentration of chlorophylls in the flavedo at immature stages and their degradation as ripening progresses could lead to a higher availability of free phytol to form PPP, higher influx into the tocopherol-core pathway and an enhancement of tocopherol contents. Accumulation of tocopherols concomitantly with chlorophylls breakdown during color change has been also described in fruit of other species like pepper (Osuna-García et al., 1998; Koch et al., 2002) and olive (Georgiadou et al., 2016).

The transcriptional analysis of tocopherol genes revealed that differences in tocopherol concentrations between tissues were associated with a generalized higher expression in the flavedo than in the pulp (Figures 4–7), which reinforces the hypothesis of the differential regulation of tocopherols accumulation between tissues. Moreover, transcriptional patterns during fruit maturation and correlation analysis pointed specific candidate genes that may regulate tocopherol levels in each tissue (Figures 4–8). In the flavedo, *TAT1* and *VTE4* showed a significant positive correlation to total, α- and γ-tocopherol contents (Figures 8A,B) suggesting the importance of these genes in regulating tocopherol accumulation in this tissue. *TAT1* plays a major role in tocopherol synthesis by regulating HGA availability (Riewe et al., 2012), and an induction of this gene during senescence has been previously reported in Arabidopsis leaves, and associated with an increase in α- and γ-tocopherol content (Holländer-Czytko et al., 2005). On the other hand, the gene *VTE4*, which encodes for γ-TMT, plays a role in shaping tocopherol composition rather than increasing tocopherol contents (Bergmüller et al., 2003). Modifications in the expression of *VTE4* have been successful in increasing α-tocopherol but in detriment of γ-tocopherol contents, and thus not altering total contents (Bergmüller et al., 2003; Collakova and DellaPenna, 2003a; Maeda et al., 2006). Therefore, in the flavedo of citrus the combined up-regulation of *TAT1* and *VTE4* during maturation could indicate a higher influx into the tocopherol core-pathway, due to a higher availability of HGA, which afterward is mostly converted into α-tocopherol by the increase in downstream *VTE4*. Still, it is important to keep in mind that many substrates and enzymes involved in tocopherol synthesis also participate in other metabolic pathways, such as HGA, and therefore the possible channeling to other pathways should be considered. Additionally, other genes, such as *HPPD* or *GGPPS1*, were also induced during ripening in specific citrus species and may also contribute to the increase of tocopherols, although they were not significantly linked to tocopherol contents in the network analysis (Figure 8B).

The expression of most genes involved in the regulation of PPP production tended to decrease in the flavedo during maturation with no apparent effect on tocopherol contents (Figures 2, 4A,B). Mutant plants of Arabidopsis in some of these genes (*vte6*, *dxs*, and *ggpps11*) have resulted in a reduction in tocopherol levels (Estévez et al., 2001; vom Dorp et al., 2015; Ruiz-Sola et al., 2016). Nonetheless, the expression of *GGPPS6*, the citrus orthologous gene of Arabidopsis *GGPPS11*, was significantly and negatively correlated with total and α-tocopherol levels, whereas *VTE6* and *DXS2* were negatively correlated with γ-tocopherol (Figure 8B). A down-regulation of *DXS* has been previously reported in the flavedo of grapefruit, orange, and mandarin linked to the onset of chlorophylls degradation at color break, rather than to the accumulation of other MEP-derived isoprenoids (Alós et al., 2006; Alquézar et al., 2008, 2013; Lado et al., 2015, 2019). Collectively, these results suggest that the supply of PPP by the recycling of free phytol or by *de novo* synthesis through the MEP pathway may not constrain tocopherol synthesis in the flavedo of *Citrus* during maturation.

In the pulp, most of the genes exhibited a similar expression tendency to that of the flavedo (Figures 4–7), although tocopherol concentrations were lower and followed contrasting temporal patterns (Figures 2, 3). A similar pattern of expression among genotypes was detected in the pulp for the genes *TAT1*, *HPPD*, *VTE6*, *GGPPS1*, and *GGDR* (Figure 6), whereas changes in the expression of the other genes followed a distinct profile depending on the specie (Figures 6, 7). Interestingly, *TAT1* was induced during maturation in the four species, similarly to the changes detected in the flavedo but opposite to the pattern of tocopherol accumulation in the pulp, and *HPPD* was induced in the pulp and in the flavedo of grapefruit, orange, and mandarin (Figures 4C, 6C). An induction of genes of the SK pathway (*TAT1* and *HPPD*) has also been observed with maturation in tomato and olive fruit but, as in the citrus pulp, did not mirror the changes in tocopherol contents (Quadrana et al., 2013; Georgiadou et al., 2015). This suggests that the up-regulation of these genes could be developmentally modulated and not necessarily associated to tocopherol contents in the pulp or in fleshy fruits. Since both *TAT1* and *HPPD* are involved in the synthesis of the precursor HGA, which is not specific of tocopherol synthesis, it is likely that their induction is related to the synthesis of other tocochromanols which also use HGA as a precursor (Szymańska and Kruk, 2010; Kruk et al., 2014; Burgos et al., 2021). In relation to the other genes involved in tocopherol synthesis, the decrease in tocopherol contents observed in the pulp of grapefruit, orange, and mandarin was synchronized with the down-regulation of the genes *VTE6*, *DXS2*, *GGDR*, and *VTE2* (Figures 6, 7). These results suggest that transcriptional regulation of these genes play a role determining tocopherol content in the pulp, which was supported by the correlation network results (Figures 8C,D). These genes, in particular those involved in the supply of PPP, have been previously proposed as limiting steps in tocopherol accumulation in the flesh of tomato (Quadrana et al., 2013; Almeida et al., 2015). In the pulp of lemon, expression of these genes varied from the other species but still reflected the minor changes detected in tocopherol accumulation during ripening in this specie. In the pulp, genes *TAT1*, *HPPD*, *DXS1*, *GGPPS1*, *VTE1*, and *VTE4* showed a negative correlation with total contents or a specific tocopherol form (Figures 8C,D). Then, in this tissue, the down-regulation of genes involved in regulating PPP availability (*VTE6*, *DXS2*, and *GGDR*) may constrain tocopherol synthesis, and the up-regulation of other genes of the SK pathway (*TAT1* and *HPPD*) and tocopherol-core pathway (*VTE1* and *VTE4*) is not enough to compensate this limitation.

The network analyses in the flavedo and pulp also revealed genes that are co-regulated in both tissues during fruit maturation but in the pulp a higher number of genes were co-regulated compared to flavedo (Figures 8B,D). The gene *VTE6* was positively linked to *DXS2* and *GGDR*, all involved in the supply of PPP in both tissues. Other genes that seemed to be co-expressed in both tissues were those of the SK pathway with the genes involved in the last steps of the tocopherol-core pathway (Figure 8).

Finally, the differences in the accumulation of tocopherols detected among genotypes in each tissue (Figures 2, 3) were not clearly related to the expression of any gene (Figures 4–7; Supplementary Tables 5, 6), although some trends were observed in the flavedo. Focusing on differences in gene expression at early maturation stages, when differences among genotypes were already evident (Supplementary Table 3), a higher expression of *GGDR* and *VTE3b* was detected in the flavedo of lemon and orange than in the other genotypes (Supplementary Table 5), which could explain the higher contents in these two species. Higher expression of these genes has been previously reported in *Citrus* genotypes accumulating higher tocopherol concentrations (Rey et al., 2021a) and also in other fruit species as tomato (Quadrana et al., 2013; Gramegna et al., 2019).

## Conclusion

This study addresses for the first time a comparative analysis of tocopherols accumulation and transcriptional regulation of their biosynthetic genes during fruit maturation of four *Citrus* species. Differences in tocopherol contents were detected between the flavedo and pulp, and also during maturation and genotypes. Concentration of tocopherols in the flavedo were between 2 and 50 times higher than in the pulp, and this was associated with higher expression levels in the flavedo of most genes involved in the precursors PPP and HGA synthesis, and the tocopherol core-pathway. Moreover, tocopherols increased in the flavedo with maturation while they tended to decrease in the pulp, and correlation and network analysis allow to suggest candidate genes regulating tocopherol accumulation in each tissue. In the flavedo, the increase in tocopherol contents mirrored a marked up-regulation of *TAT1* and *VTE4*, while contents in the pulp may be limited by the expression of *VTE6*, *DXS2*, and *GGDR*, regulating PPP availability. Furthermore, the genes *TAT1* and *VTE4*, *HPPD* and *VTE1*, and *VTE6* with *DXS2* and *GGDR*, were co-regulated and shared a similar pattern during maturation in both tissues, suggesting they are developmentally modulated. Finally, the differences among genotypes were not clearly correlated with the expression of specific genes, indicating the involvement of other regulatory mechanisms modulating differences among species.

## Data Availability Statement

The original contributions presented in the study are included in the article/Supplementary Material, further inquiries can be directed to the corresponding author.

## Author Contributions

MR and LZ: conceptualization, supervision, and funding acquisition. FR, MR, and LZ: methodology and writing – review. FR: experimental work, formal analysis, data curation, and writing – original draft preparation. All authors have read and agreed to the published version of the manuscript.

## Conflict of Interest

The authors declare that the research was conducted in the absence of any commercial or financial relationships that could be construed as a potential conflict of interest.

## Publisher’s Note

All claims expressed in this article are solely those of the authors and do not necessarily represent those of their affiliated organizations, or those of the publisher, the editors and the reviewers. Any product that may be evaluated in this article, or claim that may be made by its manufacturer, is not guaranteed or endorsed by the publisher.
